# One-year prevalence of cluster headache, hemicrania continua, paroxysmal hemicrania and SUNCT in Norway: a population-based nationwide registry study

**DOI:** 10.1186/s10194-024-01738-x

**Published:** 2024-03-06

**Authors:** Knut Hagen

**Affiliations:** 1 Department of Medical Quality Registries and Clinical Research Unit, St. Olav’s University Hospital, Trondheim, 7006 Norway; 2 Department of Neuromedicine and Movement Science, NorHEAD-Norwegian Centre for Headache Research, Norwegian University of Science and Technology, Trondheim, Norway

**Keywords:** Cluster headache, Epidemiology, General population

## Abstract

**Background:**

There is lack of population-based studies evaluating the prevalence of paroxysmal hemicrania, hemicrania continua and short-lasting unilateral neuralgiform headache attacks.

**Objectives:**

The aim of this study was to investigate the gender-specific 1-year prevalence of cluster headache, paroxysmal hemicrania, hemicrania continua, and short-lasting unilateral neuralgiform headache attacks.

**Methods:**

A nationwide study was conducted from January 1 2022 and December 31 2022 by linking diagnostic codes from Norwegian Patient Registry and prescription of relevant drugs from Norwegian Prescription Database on an individual basis. The 1-year prevalence with 95% confidence intervals (CI) of cluster headache, paroxysmal hemicrania, hemicrania continua and short-lasting unilateral neuralgiform headache attacks are estimated based on the combination of diagnostic codes, prescription of drugs and corresponding reimbursement codes.

**Results:**

Among 4,316,747 individuals aged ≥ 18 years, the 1-year prevalence per 100,000 was 14.6 (95% CI 13.5–15.8) for cluster headache, 2.2 (95% CI 1.8–2.7) for hemicrania continua, 1.4 (95% CI 1.0–1.8) for paroxysmal hemicrania, and 1.2 (95% CI 0.8–1.4) for short-lasting unilateral neuralgiform headache attacks. For all the trigeminal autonomic cephalalgies, cluster headache included, the prevalence was higher for women than men.

**Conclusions:**

In this nationwide register-based study, we found a 1-year prevalence per 100,100 of 14.6 for cluster headache, 2.2 for hemicranias continua, 1.4 for paroxysmal hemicranias, and 1.2 for short-lasting unilateral neuralgiform headache attacks. This is the first study reporting higher prevalence of cluster headache for women than men.

## Introduction

Four different trigeminal autonomic cephalalgies (TACs) are defined in the third edition of International Classification of Headache Disorders, all strict unilateral primary headaches associated with ipsilateral autonomic features [1]. Cluster headache (CH) is most common of the TACs with a median lifetime prevalence of 121 per 100,000 [2, 3]. The corresponding 1-year prevalence in adults varies between 30 and 150 per 100,000 [4–6]. Very little is known about the prevalence of paroxysmal hemicrania, hemicrania continua and short-lasting unilateral neuralgiform headache attacks with conjunctival injection and tearing (SUNCT) or with cranial autonomic symptoms (SUNA). In the Vågå study of Norway including 1838 adult inhabitants, two individuals (0.1%) with SUNCT like headache was identified, whereas probable hemicrania continua found in one individual (0.05%), although no indomethacin test was performed [7]. There is no available updated information about population-based prevalence of paroxysmal hemicrania, hemicrania continua and SUNCT/SUNA [8, 9].

The primary aim of this study was to investigate the gender-specific 1-year prevalence of cluster headache, paroxysmal hemicrania, hemicrania continua, and SUNCT/SUNA in Norway.

## Methods

### Study design

This is a nationwide register-based Norwegian study using data from the Norwegian Patient Registry (NPR) and the Norwegian Prescription Database (NorPD) linked on an individual level.

### Study population

The present study include the whole population of Norway aged ≥ 18 years (in total 4,316,747 inhabitants; 2,148,440 women and 2,168,307 men), and data were collected from January 1, 2022 to December 31, 2022.

### Data sources

NPR and NorPD were linked on an individual level by a unique 11-digit person number. The NPR contains individual-level specialist health care data on diagnostic codes for the conditions using the International Classification of Diseases 10th Revision (ICD-10) codes relevant for the treatment that has been provided to the patient [3, 10]. In the present study, data from NPR included the ICD-10 codes G44.0 and G44.8, sex and county of residence.

NorPD contains information on an individual level of all prescribed drugs dispensed to individuals in ambulatory care irrespective of public reimbursement [3, 11]. The data from NorPD included age and drug information including dosage, package size, and brand name, and reimbursement codes for the following restricted ACT codes: NO2C (triptans and calcitonin gene-related peptide monoclonal antibodies (GCRP mAbs)), M01AB01 (indomethacin), N03AX09 (lamotrigine), C08DA01 (verapamil), N03AX11 (topiramate) and N05AN01 (lithium).

The validity of the codes G44.0 and G44.8 from the NPR has not been evaluated previously. Thus, before the present study was done, a validation study was performed in new national quality register for severe headaches in Norway [12]. The diagnosis codes G44.0 and G44.8 sent to NPR was compared to diagnosis based on reviewing headache information in 302 medical records at four different hospitals in middle of Norway performed by a headache expert. Compared to diagnose based on medical record, the positive predictive values of G44.0 was 79% (95% CI 74–85%). For the NRP code G44.8, 24% (95% CI 16–32%) had hemicrania continua or SUNCT/SUNA according to medical records, the remaining 76% other headache diagnoses [12].

### Diagnostic definition

The diagnoses of respectively paroxysmal hemicrania (ICD-10 code G44.0) and hemicrania continua (ICD-10 code G44.8) required at least two prescriptions and ≥ 200 tablets of Indomethacin. Use of a specific reimbursement for chronic pain (code -71) confirmed the diagnoses. The diagnosis of SUNCT/SUNA (ICD-10 code G44.8) required at least two prescriptions and ≥ 200 tablets of lamotrigine without use of reimbursement code G40 for epilepsy. The diagnosis of cluster headache (ICD-10 code G44.0) required prescription of triptans with the reimbursement code G44.0 or N90 according to the International Classification of Primary Care version 2 separately or in combination with prescription with respectively verapamil, lithium, topiramate, and /or CGRP mAbs. The reimbursement codes were controlled for all individuals with prescription of preventive drugs without prescription of any triptans. Persons with other reimbursement codes than G44.0 or N90 (e.g., I10-I25 hypertension and angina pectoris for verapamil and F3 affective disorders for lithium) were excluded.

### Statistics

Mean age and age range was presented for all included individuals separated by diagnostic status. The 1-year prevalence with 95% confidence intervals (CI) [13] was presented by gender and female/male ratio, and by health region with north/east ratio. For cluster headache, the prescription of acute and preventive medication was presented with the number of individuals and percent. Data analyses were performed with the IBM SPSS version 29 (SPSS, Chicago, Illinois, USA).

### Ethics

Ethical approval and waiver of the requirement for obtaining patient consent were granted by the Regional Committee for Medical Research (REK 2022/ 559,971), by the Norwegian Directorate of Health (23/930–4), and by Directorate of eHealth (2023/H55).

## Results

Among the 4,316,747 inhabitants of Norway aged ≥ 18 years, 971 individuals (22.5 per 100 000/year) were registered with ICD-10 diagnosis code G44.0 in NPR in the period between from January 1, 2022 to December 31, 2022, whereas 1,150 (26.6 per 100 000/year) had G44.8. Among these, 213 persons (22%) with G44.0 and 641 persons (56%) with G44.8 did not have any prescription of relevant drugs in NorPD during 2022 (Table 1). The remaining 1267 persons had at least one prescription (in total 13,117 prescriptions, mean of 10.4 prescriptions per person, range 1–313).

**Table 1:** Demographic factors related to headache diagnosis and prescription status

Status based on prescription	Number of men (%)	Total number	Age, mean (SD) and age range
Cluster headache	284 (45%)	630	45.6 (14.4) 18–88
Paroxysmal hemicrania	11 (19%)	59	45.9 (14.6) 20–83
Hemicrania continua	27 (28%)	97	47.1 (13.9) 18–84
SUNCT/SUNA	16 (36%)	45	51.3 (17.9) 18–84
No TACs based on prescriptions	88 (24%)	356	44.5 (14.4) 18–88
One indomethacin prescription (test dose)	30 (38%)	80	44.9 (15.9) 19–87
G44.0 No prescription	No data	213	46.1 (16.2) 18–89
G44.8 No prescription	No data	641	49.2 (16.5) 18–94

Totally 630 individuals got the diagnosis of cluster headache, 59 paroxysmal hemicranias, 97 hemicrania continua and 45 SUNCT/SUNA. The corresponding 1-year prevalence per 100,000 was 14.6 (95% CI 13.5–15.8) for cluster headache, 2.2 (95% CI 1.8–2.7) for hemicrania continua, 1.4 (95% CI 1.0–1.8) for paroxysmal hemicrania, and 1.2 (95% 0.8–1.4) for SUNCT/SUNA (Table 2). For all headache diagnoses, the prevalence was higher for women than men with a women/men ratio between 1.2 and 4.4 (Table 2). The 1-year prevalence was higher in Northern Norway compared to eastern Norway with a North/East ratio between 1.3 and 2.6 (Table 2).

**Table 2:** One-year prevalence per 100,000 with 95% CI of cluster headache, paroxysmal hemikrania, hemicrania continua and SUNCT/SUNA separated by gender and health regions in Norway

Gender and region of Norway	Cluster headache	Paroxysmal hemicrania	Hemicrania continua	SUNCT/SUNA
Both gender	14.6 (13.5–15.8)	1.4 (1.0–1.8)	2.2 (1.8–2.7)	1.2 (0.8–1.4)
Men	13.1 (11.6–14.7)	0.5 (0.3–0.9)	1.2 (0.8–1.8)	0.7 (0.4–1.2)
Women	16.1 (14.5–17.9)	2.2 (1.7–3.0)	3.3 (2.6–4.1)	1.3 (0.9–2.0)
Women/Men ratio	1.2	4.4	2.8	1.9
Northern Norway	19.3 (15.3–24.2)	0.8 (0.2–2.4)	4.5 (2.8–7.3)	2.3 (1.1–4.4)
Middle of Norway	14.8 (11.9–18.3)	2.7 (1.6–4.6)	3.3 (2.0–5.2)	1.4 (0.6–2.8)
West coast	12.2 (10.1–14.8)	2.0 (1.2–3.2)	1.6 (1.0–2.8)	0.7 (0.3–1.5)
Eastern Norway	14.7 (13.2–16.3)	0.5 (0.1–1.4)	1.9 (1.4–2.5)	0.9 (0.6–1.4)
North/East Ratio	1.3	1.6	2.4	2.6

Among the 630 patients with cluster headache, 202 (32%) had prescription of triptans without any use of preventive medication (Table 3). Regarding prescription of preventive medication, verapamil was the most common (43%), whereas very few had prescription of lithium (5%) (Table 3).

**Table 3:** Prescription of drugs among 630 individuals with cluster headache

Prescriptions of drugs	Number
Triptans	
Any type	523 (83%)
Injection	371 (59%)
Nasal spray	215 (34%)
Tablets in combination with of preventive medication	30 (5%)
Injection and/or nasal spray in monotherapy without use of preventive medication	202(32%)
Preventive drugs	
Verapamil	273 (43%)
CGRP mAbs	128 (20%)
Topiramate	99 (16%)
Lithium	30 (5%)
Prescription of ≥ 1 preventive drugs	431 (68%)
Prescription of ≥ 2 preventive drugs	188 (30%)
Prescription of ≥ 3 preventive drugs	21 (3%)

## Discussion

This is the first nationwide register-based study presenting 1-year prevalence of respectively hemicrania continua, paroxysmal hemicrania and SUNCT/SUNA. This is also the first study reporting higher prevalence of cluster headache for women than men.

### Comparison with other studies

The estimated 1-year prevalence of 14.6 per 100,000 of cluster headache was much lower than the corresponding 1-year prevalence of 119 and 150 per 100,000 reported in two German studies from 2007 [5, 6], and also lower than 30 and 77 per 100,000 found in Ethiopia in 1995 [4]. In contrast to the register-based design of present study, a combination of questionnaire, telephone- and face-to-face interviews were used in these studies (4–6). As expected, our 1-year prevalence was higher than the estimated incidence of 3.0 per 100,000/year in the previous Norwegian register-based study with similar design with data from the period between 2008 to 2016, and lower than the reported lifetime prevalence of 48.6 per 100,000 [3]. They found a relatively low male-to-female ratio of 1.5 compared to previous studies [3]. Interestingly, we have as the first study found a higher prevalence of cluster headache in women than men.

There is a lack of previous large-scale epidemiological studies estimating the prevalence of paroxysmal hemicrania, hemicrania continua, and SUNCT/SUNA. The Vågå study performed in the period between 1995 and 1997 estimated a very high lifetime prevalence per 100,000 of respectively of 108 with SUNCT like headache and 54 with probable hemicrania continua [7]. However, drug treatment was not done for the few individuals the lifetime estimates were based on [7]. In the present study, we found a much lower 1-year prevalence of respectively 2.2 for hemicrania continua and 1.2 for SUNCT/SUNA.

The occurrence of TACS tended to be higher in North comperes to east of Norway. No previous studies have evaluated such impact of North versus South for TACs.

In accordance with the present study, verapamil was the most prescribed preventive drug and lithium prescribed in very few in the previous register-based study from Norway [3]. In contrast to the previous study, we found that CGRP mAbs were the second most prescribed preventive medication in patients with cluster headache, most likely reflecting that Galcanezumab has been approved for episodic cluster headache in Unites States [14]. The vast majority of cluster patients had repeated prescriptions of Galcanezumab or other CGRP mAbs using the reimbursement code for chronic migraine, since CGRP mAbs are not reimbursed for the treatment cluster headache in Norway.

### Interpretation

Our 1-year prevalence is a minimum estimate and most likely underestimating the real prevalence, because individuals with episodic types of TACS without attacks during 2022 will not be included in the present study. On the other hand, individuals with cluster headache without drug prescription (e.g. those using oxygen as acute treatment and/or treatment with e.g. nerve blocks) have been registered in the NPR with code G44.0. If we included all the 213 individuals with G44.0 in NPR without any drug prescription, the 1-year prevalence of cluster headache would have increased from 14.6 to 19.5 per 100, 000.

For the first time, we found a female predominance of cluster headache with a female-male ratio of 1.2. This can partly be explained by difference in seeking healthcare between genders. According to updated data from Statistics Norway, women seek healthcare more frequently, are more likely to suffer from migraine and to be referred to specialist [15]. Because comorbidity of cluster headache and migraine is common, we may suggest that women may be more likely to get the diagnosis of cluster headache and other types of TACS compared to men. In addition, women are more predisposed for cluster headache because of change in smoking habits during the last decades. The prevalence of daily smokers, a known risk factor of cluster headache [16], has become similar for men and women in Norway [15].

The occurrence of TACS tended to be higher in North comperes to east of Norway. This is not explained by access to neurologist, because such access is much better in eastern Norway compared to Northern Norway. Speculatively, this may be related to season variation in North including a long dark season in the winter followed by bright daylight and midnight sun in the summer period. Interestingly, this light season has previously been found to be a trigger for migraine [17]. Accordingly, some impact of midnight sun on TACs may be suggested.

In the previous published European treatment guidelines for cluster headache [18] and in the updated Norwegian guidelines, lithium and topiramate are both listed as second alternatives to verapamil. The low use of lithium can be explained by the need of careful monitoring of the patient and frequent occurrence of adverse events [14].

### Strengths and limitations

The major strengths of the present study is the large-scale nationwide design using well-established registers, making it possible to estimate updated data of the minimum 1-year prevalence with the need of treatment with drugs in respectively cluster headache, paroxysmal hemicrania, hemicrania continua, and SUNCT/SUNA. The validity of NPR diagnostic codes G44.0 and G44.8 applied was not optimal [12], but reimbursement codes in NorPD were evaluated before the final diagnoses were made.

We may have missed several individuals with TACs because a limited number of drugs were order from NorPD. All 13,117 prescriptions were evaluated manually before the diagnoses were determined. Thus, we only ordered first choice drug for hemicrania continua, paroxysmal hemicrania, and SUNCT/SUNA. On the other hand, many different drugs were available for cluster headache. However, melatonin, prednisolone and opioids were not included in the order from NorPD.

Finally, we were not able to differentiate between episodic and chronic forms of the four TACs. The present study had waiver of the requirement for obtaining patient consent, and clinical interviews and investigations could not be done. Due to these lacks, misdiagnoses cannot be ruled out. Migraine with autonomic symptoms is one possible misdiagnosis. On the other hand, neurologists in Norway have become more aware of the diagnostic criteria of TACS because these may be patients included new national quality register for severe headaches in Norway [12]. Although 1-year prevalence studies will be criticized for underestimating the real prevalence [3], the study is informative for clarifying the workload in the public health care system during a year. In the present study, signed consent was obtained.

## Conclusions

In this nationwide register-based study, we found a 1-year prevalence per 100,100 of 14.6 for cluster headache, 2.2 for hemicranias continua, 1.4 for paroxysmal hemicranias, and 1.2 for SUNCT/SUNA. This is the first study reporting higher prevalence of cluster headache for women than men.

## Data Availability

No datasets were generated or analysed during the current study.

## Abbreviations

CI: Confidence interval
ICD-10: International Classification of Diseases 10th Revision
SUNCT: Short-lasting unilateral neuralgiform headache attacks with conjunctival injection and tearing
SUNA: Short-lasting unilateral neuralgiform headache attacks with cranial autonomic symptoms
TACs: Trigeminal autonomic cephalalgies
